# Human Senataxin Resolves RNA/DNA Hybrids Formed at Transcriptional Pause Sites to Promote Xrn2-Dependent Termination

**DOI:** 10.1016/j.molcel.2011.04.026

**Published:** 2011-06-24

**Authors:** Konstantina Skourti-Stathaki, Nicholas J. Proudfoot, Natalia Gromak

**Affiliations:** 1 Sir William Dunn School of Pathology, University of Oxford, South Parks Road, Oxford, OX1 3RE, UK

## Abstract

We present a molecular dissection of pause site-dependent transcriptional termination for mammalian RNA polymerase II (Pol II)-transcribed genes. We show that nascent transcripts form RNA/DNA hybrid structures (R-loops) behind elongating Pol II and are especially prevalent over G-rich pause sites positioned downstream of gene poly(A) signals. Senataxin, a helicase protein associated with AOA2/ALS4 neurodegenerative disorders, acts to resolve these R-loop structures and by so doing allows access of the 5**′**–3**′** exonuclease Xrn2 at 3**′** cleavage poly(A) sites. This affords 3**′** transcript degradation and consequent Pol II termination. In effect, R-loops formed over G-rich pause sites, followed by their resolution by senataxin, are key steps in the termination process.

## Graphical abstract

**Graphical Abstract:**

**Figure:**
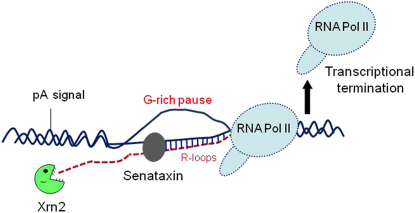

**Highlights:**

► Senataxin is required for pause-dependent Pol II termination in human genes ► R-loops accumulate over G-rich pause regions and are required for termination ► R-loops dependent on transcription, functional poly(A) signal, and pause elements ► Senataxin resolves R-loops to allow Xrn2 to mediate Pol II release from the gene

## Introduction

All steps in gene expression are thought to be interconnected and co-ordinately regulated (Moore and Proudfoot, 2009). Transcription termination is defined as the cessation of RNA synthesis and release of RNA polymerase II (Pol II) from its DNA template. Recent evidence shows that this process is important for optimal protein production (West and Proudfoot, 2009). Efficient Pol II termination is dependent on both a functional poly(A) signal and downstream terminator sequences (Connelly and Manley, 1988; West et al., 2004, 2008; Whitelaw and Proudfoot, 1986). Two classes of terminator sequences have been identified in human genes: cotranscriptionally cleaved (CoTC) RNA sequences and transcription pause sites. The human β-globin gene contains a CoTC element, located ∼1kb downstream of the poly(A) site, which is essential for efficient Pol II transcriptional termination (Dye and Proudfoot, 1999, 2001). CoTC RNA undergoes rapid cleavage that exposes the 3′-flanking RNA to degradation by the 5′–3′ exonuclease Xrn2 (West et al., 2004). The function of Xrn2/Rat1 in the termination process has been studied quite extensively (Kaneko et al., 2007b; Kim et al., 2004; Luo et al., 2006; Xiang et al., 2009). Alternatively, transcription pause sites act to slow down elongating Pol II and have been identified in α2- and γ-globin genes (Enriquez-Harris et al., 1991; Plant et al., 2005). Another pause site, defined as the G-rich MAZ termination element, facilitates termination of the human C2 complement gene (Ashfield et al., 1994). The human β-actin gene also possesses a G-rich pause element downstream of the poly(A) signal, which similarly acts to promote transcriptional termination through Xrn2-mediated degradation of the downstream transcript exposed by poly(A) site cleavage (Gromak et al., 2006). Interestingly, genome-wide analysis reveals that G-rich sequences immediately downstream of poly(A) signals are relatively common in mammalian genes (Salisbury et al., 2006), suggesting that these elements may play an essential function in gene regulation.

Sen1, an RNA/DNA helicase, is known to cooperate with Xrn2/Rat1 to promote efficient transcriptional termination in *S. cerevisiae* (Kawauchi et al., 2008). In addition a single amino acid mutation in the Sen1 helicase domain compromises its function, resulting in altered genome-wide distribution of Pol II over both coding and noncoding genes (Steinmetz et al., 2006). Sen1 is also involved in the processing of tRNA, snRNAs, and snoRNAs, as well as in transcription-coupled DNA repair (Rasmussen and Culbertson, 1998; Steinmetz et al., 2006; Ursic et al., 1997). Furthermore, biochemical studies have revealed that Sen1 N-terminal domain interacts with Rpb1, the largest Pol II subunit; Rad2, required for DNA repair; and Rnt1 (RNase III), essential for the maturation of multiple RNAs (Ursic et al., 2004).

Human senataxin, the mammalian Sen1 homolog, was initially identified when mutations causing ataxia oculomotor apraxia 2 (AOA2) and amyotrophic lateral sclerosis type 4 (ALS4) were mapped to the senataxin gene (SETX) (reviewed in James and Talbot, 2006; Palau and Espinós, 2006). These diseases result in progressive degeneration of motor neurons in the brain and spinal cord, progressive muscle weakness, and atrophy. SETX encodes a 302.8 kD widely expressed protein containing an N-terminal putative protein-protein interaction domain and a C-terminal DEAD-box helicase domain, followed by a nuclear localization signal (NLS) (Chen et al., 2004). Most senataxin mutations found in AOA2/ALS4 families either cause premature protein termination or interfere with the function of the helicase or N-terminal protein interaction domains (Chen et al., 2004; Criscuolo et al., 2006; Duquette et al., 2005; Fogel and Perlman, 2006; Moreira et al., 2004). However, the precise mechanism of toxicity caused by these mutations and manifested in AOA2/ALS4 patients remains to be elucidated. Since senataxin localizes to both nucleus and cytoplasm, it may possess additional cytoplasmic roles in RNA metabolism and translational regulation (Chen et al., 2006). The domain structure of senataxin is similar to Sen1, and its C-terminal helicase domain is also homologous to helicase domains of human immunoglobulin μ-Binding Protein 2 (IGHMBP2) and the nonsense-mediated mRNA decay factor (UPF1). Interestingly, mutations in IGHMBP2 have been previously found to cause autosomal recessive spinal muscular atrophy (SMA) (Grohmann et al., 2001). At a cellular level, mutations in senataxin cause hypersensitivity to single-strand DNA damage, but not to ionizing radiation, as observed in ataxia-telangiectasia mutated (ATM)-deficient cells (Chen et al., 2006; Suraweera et al., 2007). Similarly to its yeast counterpart, senataxin interacts with Pol II and other RNA processing factors, such as poly(A)-binding proteins 1 and 2 (PABP1/2), hnRNPs, SAP155, and SMN, pointing toward a role for senataxin in the regulation of gene expression (Suraweera et al., 2009).

We describe here the role of senataxin in the regulation of gene transcription. We demonstrate that senataxin depletion in HeLa cells causes an increase in readthrough RNA and Pol II density downstream of the poly(A) site, implying an involvement in transcriptional termination. Using DNA immunoprecipitation (DIP) analysis on endogenous genes and transfected constructs, we demonstrate that RNA/DNA hybrid structures (R-loops) are formed across transcription units by interaction of the nascent transcript with the ssDNA template behind the elongating Pol II. R-loops are dependent on transcription, functional poly(A) signals, and termination elements. Overexpression of RNase H1 in vivo acts to resolve R-loops and causes transcriptional readthrough. Upon senataxin depletion, R-loops are stabilized downstream of the poly(A) signal, preventing Xrn2 recruitment, nascent RNA degradation, and efficient termination. We predict that senataxin acts to resolve R-loops in pause-dependent transcription termination.

## Results

### Senataxin Promotes Transcriptional Termination on Transfected Gene Constructs

**Figure 1:**
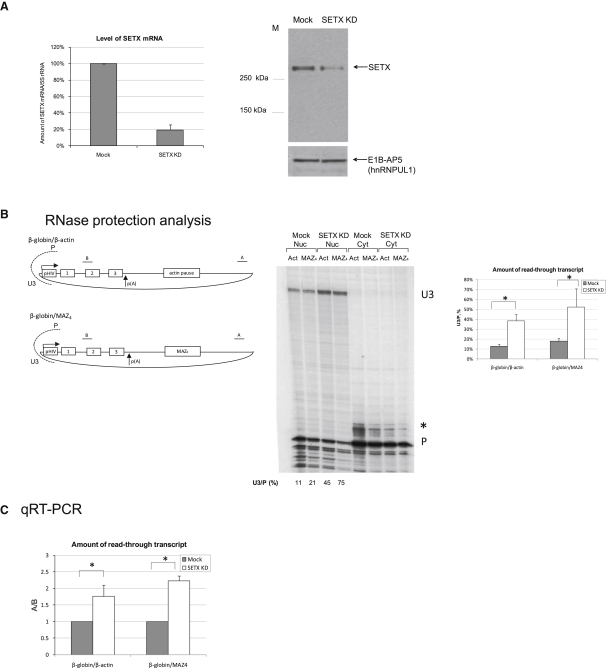
Transcriptional Termination Defect of Constructs with G-Rich Pause Sites in Senataxin-Deficient HeLa Cells (A) RNAi-mediated depletion of human senataxin in HeLa cells. Left panel: qRT-PCR analysis of mock-treated and senataxin siRNA1-treated HeLa cells. Bars represent average values ± SD from three independent biological experiments. 5S rRNA was used as a control. The amount of SETX mRNA in mock cells was taken as 100%. Right panel: western blot analysis of 60 μg of protein extracts from mock-treated and senataxin siRNA1-treated HeLa cells using anti-senataxin antibody. E1B-AP5 protein (∼120 kDa) was used as a loading control. Cut-out middle lane of this gel contained lower amount of protein extract. (B) Left panel: diagram of β-globin/β-actin and β-globin/MAZ4 gene constructs, with exons shown as boxes and pause elements indicated. Poly(A) signal is denoted by arrows. P/U3 probe was used in RNase protection analysis and is shown as dashed lines. “A” and “B” in diagram denote positions of PCR products used to calculate the amount of readthrough transcript in (C). Middle panel: RNase protection analysis of β-globin/β-actin and β-globin/MAZ4 constructs from mock-treated and senataxin siRNA1-treated cells performed with P/U3 riboprobe. 85 nt fragment, “P,” derives from transcripts initiated on the HIV promoter, while an ∼240 nt band (U3) derives from readthrough transcription. ^∗^ denotes partial RNase digestion product detected in the cytoplasm in some experiments. Right panel: readthrough transcript level was calculated as a signal ratio of U3/P (%), based on three independent experiments; values are ±SD. (C) Termination efficiency of β-globin/β-actin and β-globin/MAZ4 constructs from mock-treated and senataxin siRNA1-treated cells using qRT-PCR analysis on the total RNA. Readthrough transcript level was calculated as a ratio of product A (readthrough RNA) versus product B (gene transcript) from three independent experiments; values are ±SD. The A/B ratio in mock-treated cells was taken as 1. ^∗^ indicates statistical significance (p < 0.05), based on unpaired, two-tailed distribution Student's t test.

Previous work in *S. cerevisiae* has demonstrated that Sen1 is required to promote Pol I and Pol II transcriptional termination (Kawauchi et al., 2008). To test if human senataxin protein is also involved in transcriptional termination, we employed RNAi to deplete senataxin in HeLa cells to a 15% level, using SETX siRNA1 (Figure 1A). Next, we tested if the transcriptional termination profile of gene constructs transiently transfected into HeLa cells is affected in senataxin-depleted cells. Here, we used two previously described gene constructs containing the β-actin pause element or MAZ4 pause sequences, cloned downstream of the β-globin poly(A) signal instead of its termination CoTC element (Gromak et al., 2006) (Figure 1B). Transcription of these gene constructs is driven by the efficient HIV promoter, which is fully activated by coexpression of TAT *trans*-activator. Both the β-actin pause element and MAZ4 G-rich pause sequences have been previously shown to promote poly(A) site- and Xrn2-dependent transcriptional termination (Gromak et al., 2006).

To investigate if senataxin affects transcriptional termination of β-globin/β-actin and β-globin/MAZ4 constructs, we performed an RNase protection analysis (RPA) on nuclear and cytoplasmic RNA harvested from mock-treated and senataxin- siRNA1-treated cells, detecting nascent transcripts that readthrough the normal termination signals (Ashe et al., 1995; Plant et al., 2005). In these experiments we used uniformly labeled P/U3 riboprobe positioned over the HIV promoter (Figure 1B). In the nucleus it detects an 85 nt band (P), resulting from protection of correctly initiated transcripts, and a 240 nt readthrough band (U3), representing transcripts derived from nonterminated Pol II complexes that have transcribed around the plasmid. No readthrough RNA, as represented by the U3 band, was detected in the cytoplasm, reflecting a lack of its nuclear export to the cytoplasm or its cytoplasmic instability. The signal ratio between U3 and P products is indicative of the termination efficiency of Pol II on a particular construct. As demonstrated in Figure 1B (middle and right panels), senataxin depletion causes a 2.5- to 4-fold increase in the amount of the readthrough U3 band for both β-globin/β-actin and β-globin/MAZ4 constructs, suggesting a role for senataxin in transcriptional termination.

The termination efficiency of β-globin/β-actin and β-globin/MAZ4 constructs in senataxin-deficient cells was also assessed using quantitative RT-PCR analysis. In this experiment, we measured the amount of readthrough transcript A and the amount of correctly initiated transcript B (Figure 1B for primer positions). The ratio of A/B represents the efficiency of transcriptional termination. As demonstrated in Figure 1C, senataxin depletion causes a 1.8- to 2.2-fold increase in the amount of the readthrough transcript A for both β-globin/β-actin and β-globin/MAZ4 constructs, further pointing toward a role for senataxin in transcriptional termination. To avoid potential off-target effects during the RNAi procedure, we also used SETX siRNA2, targeting a different region of SETX mRNA. Both qRT-PCR and RPA revealed a transcriptional termination defect of β-globin/MAZ4 construct in SETX siRNA2-depleted cells (Figure S1).

### R-Loop Formation on Transfected Gene Constructs

The majority of senataxin mutations observed in AOA2/ALS4 patients are detected within the helicase or N-terminal protein interaction domains, suggesting their importance in senataxin function. Furthermore, the function of yeast Sen1 in transcription is likely to be associated with Sen1 RNA/DNA helicase activity, which acts to resolve RNA/DNA hybrids formed between nascent RNA and template DNA strand behind elongating Pol II (Mischo et al., 2011).

**Figure 2:**
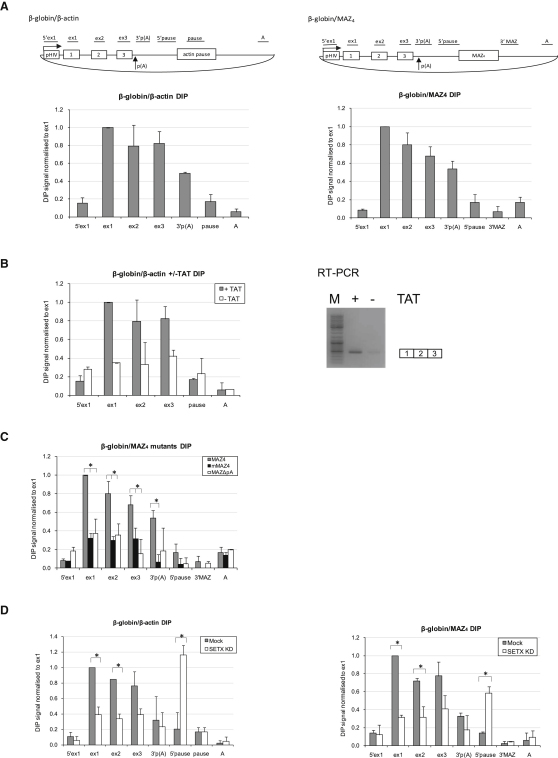
R-Loops Form over Transfected Gene Constructs and Depend on Transcription, Functional Poly(A) Signal, and G-Rich Pause Elements (A) Top panels: diagram of β-globin/β-actin (left) and β-globin/MAZ4 (right) gene constructs, with positions of primers used in DIP analysis indicated. Bottom panels: DIP analysis of β-globin/β-actin (left) and β-globin/MAZ4 (right) constructs. DIP signal was normalized to exon 1 signal. (B) R-loops are dependent on active transcription. Left panel: R-loop profile of cells transfected with β-globin/β-actin gene construct ± *trans*-activator TAT. Right panel: RT-PCR analysis of the spliced mRNA detected from HeLa cells transfected with β-globin/β-actin construct ± TAT. “M” indicates the molecular weight DNA markers. DIP signal was normalized to exon 1 signal of β-globin/β-actin construct cotrasfected with TAT. (C) R-loop profile of β-globin/MAZ4 (gray bars), mMAZ4 (black bars), and MAZ4ΔpA (white bars) gene constructs. DIP signal was normalized to exon 1 signal of β-globin/MAZ4 construct. (D) R-loop profile of β-globin/β-actin (left panel) and β-globin/MAZ4 (right panel) gene constructs in mock-treated (gray bars) and senataxin siRNA1-treated (white bars) cells. DIP signal was normalized to exon 1 signal of β-globin/β-actin (left panel) and β-globin/MAZ4 (right panel) constructs in mock-treated cells. DIP profile is based on average values ± SD from at least three independent biological experiments. ^∗^ indicates statistical significance (p < 0.05), based on unpaired, two-tailed distribution Student's t test.

To determine if the molecular function of human senataxin is associated with R-loop resolution, we tested whether RNA/DNA hybrids are formed over the transcribed regions of human genes. We employed DNA immunoprecipitation (DIP), which relies on the immunoprecipitation of noncrosslinked purified RNA/DNA hybrids, using S9.6 antibody, which recognizes RNA/DNA duplexes (Boguslawski et al., 1986; El Hage et al.,2010). We demonstrate that R-loops are formed over the transcription units of β-globin/β-actin and β-globin/MAZ4 constructs, showing enrichment at the 5′ end of each transcription unit (Figure 2A). To verify that these R-loop signals are dependent on active transcription, we performed DIP analysis on HeLa cells transfected with β-globin/β-actin gene construct without the *trans*-activator TAT and observed a reduction of R-loop signal over the coding regions of the gene (Figure 2B, left panel). Some R-loop signal still remained in −TAT samples, possibly due to significant levels of “leaky” transcription, observed in HeLa cells. Thus Figure 2B (right panel) shows that reduced mRNA levels are still detected in -TAT samples. These results confirm the transcriptional dependency of R-loops.

Next, we investigated if *cis* mutations affecting transcriptional termination of β-globin/MAZ4 construct can affect the R-loop distribution profile. Here, we used constructs containing either mutated poly(A) signal (ΔpA) or mutant MAZ sequences (mMAZ4), which do not promote transcriptional pausing. Both of these mutations cause transcriptional termination defects (Gromak et al., 2006). Interestingly, both mutations decreased the levels of R-loop signal across the β-globin/MAZ4construct (Figure 2C). Our previous studies show that strong termination defects, as observed for these constructs, cause a partial reduction in levels of transcription (West and Proudfoot, 2009) with a consequent decrease in R-loop formation. This effect is similar to that seen for the construct transfected without TAT (Figure 2B). The fact that poly(A) signal and termination pause elements are both associated with efficient transcriptional termination and the formation of R-loops implies that R-loops represent a feature of properly defined transcription units, containing functional promoter and terminator elements.

We finally tested if R-loops formed over the transfected gene constructs are dependent on senataxin. Following RNAi-mediated knockdown of senataxin, we observed a decrease in R-loops over the coding regions of the gene constructs, but their substantial increase upstream of the β-actin and MAZ4 pause elements (Figure 2D). The reduction of R-loops over the ORF is most probably due to the reduced level of transcription detected upon senataxin depletion (see Figures 4B and 4C). However, the increase in R-loops downstream of the poly(A) signal suggests a role of senataxin in their resolution that may be connected to transcriptional termination.

### RNase H Resolves R-Loops and Causes Transcriptional Readthrough In Vivo

**Figure 3:**
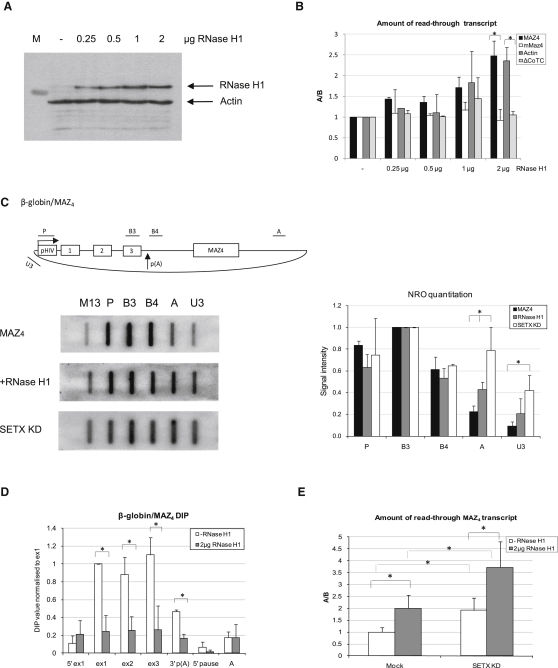
RNase H1 Resolves R-Loops and Promotes Transcriptional Readthrough (A) Western blot analysis of 30 μg of protein extracts obtained from HeLa cells transfected with increasing amounts (0–2 μg) of GFP-RNase H1 expression plasmid. Western blot was analyzed using anti-GFP antibody. Actin was used as loading control. (B) Amount of readthrough transcript detected in β-globin/β-actin, β-globin/MAZ4, β-globin/mMAZ4, and ΔCoTC constructs in mock HeLa cells (sample “-”) and HeLa cells, cotransfected with increasing amounts of GFP-RNase H1 plasmid. Readthrough transcript level was calculated as a ratio of A/B, using qRT-PCR analysis (see Figure 1B for primer positions). The A/B ratio in mock-treated cells (sample “-”) was taken as 1. This data is also presented as values for transcripts “A” and “B” separately, normalized to 5S rRNA (Figure S2). (C) Top panel: diagram of β-globin/MAZ4 gene construct with positions of NRO probes indicated. Bottom left and right panels: NRO analysis of β-globin/MAZ4 construct in mock-treated cells (black bars on the right panel), HeLa cells cotransfected with 2 μg of GFP-RNase H1 (gray bars), and senataxin siRNA1-treated cells (white bars). M13 represents a background probe. Right panel: NRO signals were quantified by PhosphoImager analysis. Background (signal from probe M13) was subtracted from each hybridization signal normalized to probe B3, taken as 1. All bars represent average values ± SD from three independent biological experiments. (D) DIP analysis of β-globin/MAZ4 construct following overexpression of 2 μg of GFP-RNase H1 in HeLa cells. Positions of primers used in DIP analysis are shown in Figure 2A. DIP profiles are based on average values ± SD from three independent biological experiments. DIP signal was normalized to exon 1 signal of β-globin/MAZ4 construct without GFP-RNase H1 overexpression. (E) Amount of readthrough β-globin/MAZ4 transcript in mock-treated and senataxin-depleted HeLa cells with or without overexpression of 2 μg of GFP-RNase H1 plasmid. Readthrough transcript level was calculated as a ratio of A/B, using qRT-PCR analysis (for primer positions see Figure 1B). The A/B ratio in mock-treated cells (−RNase H1 sample) was taken as 1. Average qRT-PCR values ± SD from four independent biological experiments are presented. ^∗^ indicates statistical significance (p < 0.05), based on unpaired, two-tailed distribution Student's t test.

To investigate if R-loops are directly associated with the transcriptional termination process, we overexpressed GFP-tagged RNase H1 (Cerritelli et al., 2003), an enzyme which specifically degrades RNA in RNA/DNA hybrids, in HeLa cells. The level of RNase H1 overexpression was measured by western blot as compared to endogenous actin (Figure 3A). We then investigated the effect of RNase H1overexpression on transcriptional termination of the β-globin/β-actin and β-globin/MAZ4 constructs. As controls, we used constructs either lacking termination elements (ΔCoTC) or containing mMAZ4 (Dye and Proudfoot, 1999; Gromak et al., 2006). The termination efficiency was measured by qRT-PCR with primers positioned over regions A and B (see Figure 1B for primer positions). As demonstrated in Figures 3B and S2, progressive overexpression of RNase H1 caused an increase in the amount of readthrough transcript of β-globin/β-actin and β-globin/MAZ4, but not β-globin/mMAZ4 and ΔCoTC plasmids. This suggests a specific effect of RNase H1 on constructs containing functional terminator G-rich pause elements.

We also measured the amount of active transcription on the β-globin/MAZ4 construct in mock-treated and RNase H1-overexpressing HeLa cells, using nuclear run-on analysis (NRO) (Figure 3C). Here, we employed single-stranded antisense DNA probes (cloned into M13 phage DNA), covering β-globin/MAZ4 regions as indicated. In mock-treated HeLa cells we observed an efficient transcriptional termination of the β-globin/MAZ4 construct, as indicated by reduced signals over probes A and U3. Interestingly, overexpression of RNase H1 (2 μg of plasmid) caused a 2-fold increase in the amount of readthrough transcription, observed over probe A. We also saw a slight increase in the amount of readthrough transcription over the probe U3. In addition, we detected a substantial termination defect in cells depleted of senataxin protein, as indicated by a 3- to 4-fold increase in A and U3 signals. To further investigate the mechanistic connection between the termination defect caused by either senataxin depletion or RNase H1 overexpression, we studied the R-loop profile of the β-globin/MAZ4 construct following RNase H1 overexpression. Using DIP analysis (Figure 3D) we show that overexpression of RNase H1 in HeLa cells diminishes R-loop levels over both gene and termination regions of the β-globin/MAZ4 construct. These data suggest that R-loops are involved in the process of transcriptional termination, and their resolution by RNase H1 induces transcriptional readthrough. Next, we tested whether RNase H1 overexpression and its ability to resolve R-loops increases the termination defect, as we observed for senataxin-deficient cells. As shown in Figure 3E, RNase H1 overexpression enhanced transcript readthrough in β-globin/MAZ4 construct in both mock-treated and senataxin-depleted cells. In effect, both the presence of R-loops and their subsequent resolution are required steps in the termination process (see Discussion). Consequently, it follows that RNase H1 overexpression and senataxin have additive termination defects. These results imply that R-loops are required for transcriptional termination, as is their resolution by senataxin.

### Senataxin Is Involved in Transcriptional Initiation and Termination of the Endogenous Human β-Actin Gene

**Figure 4:**
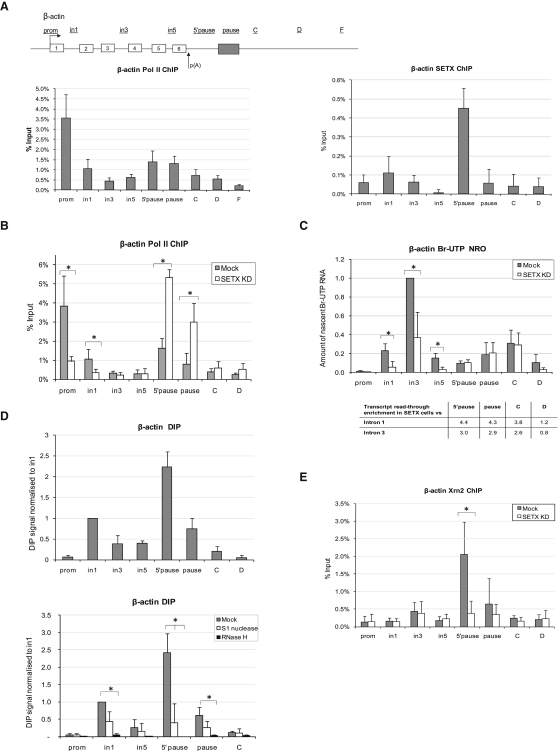
Senataxin Is Required for Pol II Transcriptional Initiation, Termination, and Xrn2 Recruitment to the Termination Regions of the Endogenous β-Actin Gene (A) Diagram of β-actin gene, with exons shown as white boxes and pause element shown as gray box. Positions of primers used in ChIP analysis are shown above the diagram. ChIP analysis was carried out with Pol II (left panel) or senataxin (right panel) antibody. (B) Pol II ChIP analysis of β-actin gene in mock-treated (gray bars) and senataxin siRNA1-treated (white bars) cells. (C) Br-UTP NRO analysis of β-actin gene in mock-treated (gray bars) and senataxin siRNA1-treated (white bars) cells. Amount of nascent Br-UTP RNA was normalized to intron 3 probe in mock-treated cells. All bars represent average values ± SD from at least three independent biological experiments. Enrichment of readthrough transcripts for probes “5′ pause,” “pause,” “C,” and “D” in senataxin-depleted cells, as compared to mock cells, was calculated relative to intron 1 and intron 3 signals. (D) Top panel: DIP analysis of β-actin gene using RNA/DNA hybrids-specific S9.6 antibody, based on average values ± SD from at least three independent biological experiments. DIP signal was normalized to intron 1 signal. Bottom panel: R-loops formed over β-actin gene are sensitive to RNase H and only partially to S1 nuclease digestion. DIP samples were either untreated (gray) or treated with 10 U of RNase H (black bars) or 200 U of S1 nuclease (white bars). (E) Xrn2 ChIP of β-actin in mock-treated (gray bars) and senataxin-depleted (white bars) HeLa cells. ChIP values are based on average values ± SD from at least three independent biological experiments. ^∗^ indicates statistical significance (p < 0.05), based on unpaired, two-tailed distribution Student's t-test.

The above experiments rely on analysis of gene constructs with different termination sequences. To validate these data using an endogenous gene system (in native chromatin), we also investigated the role of senataxin in termination of the endogenous β-actin gene in HeLa cells. In contrast to transiently transfected plasmids, transcriptional profiles of endogenous genes can be reproducibly analyzed by chromatin immunoprecipitation (ChIP) experiments, employing a number of PCR primers located over the coding and 3′-flanking regions. Previously, we have mapped a 3′-flanking pause element involved in transcriptional termination of the human β-actin gene (Gromak et al., 2006). As shown (Figure 4A, left panel), we observe Pol II enrichment over the promoter of β-actin gene and around its pause element downstream of the poly(A) signal. Accumulated Pol II levels over β-actin gene are likely to be associated with pausing at the promoter and terminator regions and are resonant with gene loop conformations (Tan-Wong et al., 2008). Pol II detected over the coding region was lower than over the promoter, suggesting that during transcription only a subfraction of Pol II molecules undergo efficient elongation as generally found for protein-coding genes (Core et al., 2008; Nechaev et al., 2010). To determine if senataxin binds the β-actin gene, we performed ChIP analysis using anti-senataxin antibody, which specifically recognizes the 303 kDa senataxin protein in HeLa cells (Figure 1A). We demonstrate that senataxin binds upstream of the β-actin pause element at the highest level (Figure 4A, right panel). The association of senataxin with β-actin gene suggests an involvement in transcriptional regulation. Therefore, we investigated the transcriptional profiles of the β-actin gene in senataxin-deficient cells. ChIP analysis revealed that, upon senataxin depletion, reduced levels of Pol II occurred over the promoter region of the β-actin gene, implying an effect on transcriptional initiation, which in turn leads to an elongation defect (Figure 4B). Additionally, we observe an increase in Pol II density over the 5′ pause and pause regions, indicative of a defect in transcriptional termination.

To further substantiate a role for senataxin in transcriptional initiation/elongation and termination, we employed NRO using Br-UTP as the labeled nucleotide (Core et al., 2008; Lin et al., 2008). Br-UTP NRO involves isolation of HeLa cell nuclei in such a way that transcribing polymerases remain actively bound to their DNA templates. These can resume transcription after addition of transcription buffer ± Br-UTP (or UTP in control samples) in vitro. The nascent RNA containing incorporated Br-UTP is selected using anti-Br-UTP antibody. The advantage of Br-UTP NRO over Pol II ChIP is the ability of Br-UTP NRO to detect actively transcribing polymerases, as opposed to total Pol II levels detected by ChIP. As demonstrated in Figure 4C, in mock-treated cells we observed low RNA signals over the promoter and intron 1 regions, due to lack of transcription over the promoter and the high level of paused Pol II accumulated over intron 1. Both of these amplicons are positioned over a known nucleosome-free region, where Pol II pauses prior to efficient transcription elongation (Egloff et al., 2009). We observed high enrichment of nascent transcripts over the intron 3 probe, positioned immediately downstream of the nucleosome-free region, indicative of efficient transcriptional elongation. Based on Br-UTP NRO, we observed a 2- to 5-fold decrease in nascent RNA levels produced over the gene body in senataxin-depleted cells as compared to mock-treated cells (Figure 4C). In contrast, we detected higher enrichment of the nascent readthrough RNA signals over the 5′ pause, pause, C, and D regions, relative to the gene body (intron 1 and 3 probes), in senataxin knockdown cells, indicating a role for senataxin in transcriptional termination (Figure 4C).

These data are consistent with our Pol II ChIP results (Figure 4B), confirming that senataxin plays a role in transcriptional regulation of human β-actin gene. Similarly, experiments in *S. cerevisiae* have shown that Sen1 is required to promote Pol I and Pol II transcriptional termination (Kawauchi et al., 2008). This points toward the generality of this senataxin-dependent termination mechanism.

### R-Loops Are Formed over Endogenous Human Genes

Next, we investigated if R-loops can be formed over the transcribed regions of endogenous β-actin gene similar to transfected gene constructs. As demonstrated in Figure 4D (top panel), R-loops were detected over the transcribed regions of endogenous β-actin gene, with their specific enrichment over the intron 1 and immediately downstream of the poly(A) signal. Interestingly, the latter region is also enriched for senataxin binding (Figure 4A, right panel). To test the specificity of the signal detected by S9.6 antibody in DIPs, we performed nuclease treatment experiments prior to the immunoprecipitation step. In particular, we treated the samples with RNase H, which recognizes R-loops, and S1 nuclease, which recognizes ssRNA and ssDNA molecules and R-loops at high enzyme concentrations. As demonstrated in Figure 4D (bottom panel), treatment of the samples with RNase H completely abolished the R-loop signals, suggesting that they are specific for RNA/DNA hybrids. As expected, R-loop signals were only partially lost by S1 nuclease treatment.

### Senataxin Is Required for Xrn2 Recruitment to Promote Efficient Transcriptional Termination

Previously, we have demonstrated the role of the human 5′–3′ exonuclease Xrn2 in the process of transcriptional termination (West et al., 2004). To confirm that Xrn2 protein binds the termination region of the endogenous β-actin gene, we performed ChIP analysis using anti-Xrn2 antibody. A specific enrichment of Xrn2 signal downstream of the poly(A) signal and upstream of the pause element of the endogenous β-actin gene was clearly evident (Figure 4E, gray bars).

Next, we tested if Xrn2 recruitment to the termination region of the endogenous β-actin gene is affected in senataxin-deficient cells. To that end, we performed Xrn2 ChIP in senataxin-depleted HeLa cells. As demonstrated in Figure 4E, Xrn2 recruitment was substantially reduced in senataxin knockdown cells. These results predict that senataxin is required for the resolution of R-loops formed downstream of the poly(A) signal, allowing Xrn2 recruitment and efficient Pol II transcriptional termination.

## Discussion

Previous studies have extensively characterized the mechanism of transcriptional termination in mammals (Kaneko et al., 2007b; Luo et al., 2006; West et al., 2004, 2008). The 5′–3′ exonuclease Xrn2 recognizes the nascent RNA cleaved at the poly(A) site, promotes its degradation, and so causes Pol II transcriptional termination. This requires termination elements located downstream of the poly(A) signal, such as pause sites or CoTC elements. Here, we describe a molecular function for human senataxin protein in this process. Using ChIP, NRO, and RPA approaches, we show that senataxin-deficient HeLa cells have transcription initiation/elongation and termination defects (Figures 1 and 4). In particular, we observe a decrease in nascent RNA and Pol II density over the gene body and an increase in the amount of readthrough RNA and Pol II density downstream of the poly(A) signal in the termination regions. We hypothesize that the function of senataxin protein in human cells may be related to its RNA/DNA helicase activity, and, in particular, its ability to unwind RNA/DNA hybrids formed behind the elongating Pol II between the nascent RNA transcript and single-stranded DNA template. Using the DIP technique, we show that R-loops can be efficiently detected over transcribed regions of the endogenous β-actin gene and transfected gene constructs (Figures 2 and 4). We observed a stronger R-loop profile over the transfected constructs than with the endogenous β-actin gene, most probably due to the high level of transcription induced by the strong HIV promoter. Furthermore, the RNA/DNA hybrid signature of R-loops was confirmed by RNase H treatment, specifically targeting their degradation (Figure 4). Based on our transfected gene constructs, we show that formation of R-loops is dependent upon active transcription, the presence of a functional poly(A) signal, and termination G-rich pause elements (Figure 2). Interestingly, senataxin depletion caused accumulation of R-loops downstream of the poly(A) signal, supporting the idea that it has a function in R-loop resolution in termination regions (Figure 2). We also demonstrate that RNase H1 overexpression in vivo resolves R-loops, resulting in transcriptional readthrough (Figure 3). In effect, R-loops formed over pause elements can be considered as an essential component of the termination process. But once they are formed, their removal by senataxin is also required. Furthermore, senataxin depletion severely affected Xrn2 recruitment to the termination regions (Figure 4).

**Figure 5:**
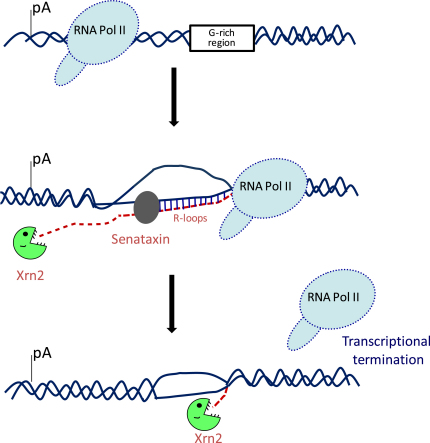
Model for Role of Senataxin and R-Loops in Transcriptional Termination DNA is shown as solid lines and RNA as a dotted line. R-loops are formed between the ssDNA template strand and nascent RNA behind the elongating Pol II. Vertical black lines denote RNA/DNA hybrids. Senataxin is shown as a gray oval over the R-loop region. R-loops formed over the G-rich pause region of human β-actin gene are necessary for Pol II to pause downstream of the poly(A) site. Senataxin is needed to resolve R-loop structures and so allow 5′–3′ exonuclease Xrn2 to degrade the nascent RNA from the site of poly(A) cleavage and catch up with paused Pol II, causing its transcriptional termination.

These results add a new molecular layer to the mechanism of Xrn2-mediated transcriptional termination in mammals (Figure 5). Overall, our results suggest that the formation of R-loops in transcriptional pause regions is essential in causing Pol II to pause downstream of the poly(A) site prior to termination. However, these R-loops have to be subsequently resolved by senataxin to release the nascent RNA and so allow its Xrn2-mediated degradation, which ultimately results in efficient Pol II transcriptional termination. The fact that R-loops play an important role in the process of transcriptional termination raises interesting issues for further investigation. In particular, formation of R-loops over the G-rich pause elements may result in folding of the G-rich nontemplate ssDNA strand into G-quadruplex structures. Indeed, genome-wide bioinformatics analysis has revealed an enrichment of G-quadruplexes in promoter and 3′UTR regions of the genes (Huppert et al., 2008). Interestingly, 3′UTR G-quadruplexes are particularly prevalent in cases where another gene was found in close proximity, suggesting that G-quadruplexes may be involved in transcriptional termination (Huppert et al., 2008).

Recent studies have demonstrated an interesting link between 3′ end processing and transcriptional initiation (Mapendano et al., 2010). In agreement with this work, our results reveal that senataxin deficiency causes a defect in both termination and initiation. This is particularly evident on the endogenous β-actin gene and less so with the transfected gene constructs, as their expression is driven by the strong HIV promoter. This further supports the idea of factors recycling due to established promoter-terminator “gene-loop” interactions in vivo. Significantly, constructs with mutations either in the poly(A) site or termination pause element displayed reduced R-loop levels. These gene constructs have a strong termination defect, with Pol II transcribing around the plasmids. While our previous work has demonstrated that both constructs display slightly reduced levels of transcription (West and Proudfoot, 2009), it is also plausible that loss of the R-loop signal may relate to loss of gene loop interactions. Whether senataxin is involved in establishing or maintaining this gene conformation will be tested in the future. Senataxin was previously found to interact with Pol II and various RNA binding factors, such as poly(A)-binding proteins 1 and 2 (PABP1/2), hnRNPs, SMN, and SAP155 (Suraweera et al., 2009). Therefore, senataxin may be involved in recognition of the poly(A) signal and/or recruitment of other transcription/RNA-processing factors. Transcriptional termination in humans requires 3′-end processing factor Pcf11, previously found to terminate stalled Pol II complexes in vitro and stimulate transcript degradation by Xrn2 in vivo (West and Proudfoot, 2008; Zhang et al., 2005; Zhang and Gilmour, 2006). Pcf11 is also implicated in the crosstalk between 3′ end processing and transcriptional initiation (Mapendano et al., 2010). It is unclear how Pcf11 is involved in senataxin-mediated R-loop resolution during transcriptional termination. Possibly, nascent RNA, released from R-loops by senataxin, provides a binding platform for Pcf11 prior to Xrn2-mediated termination.

Senataxin mutations observed in AOA2/ALS4 patients cause defects in DNA damage repair (Chen et al., 2006; Suraweera et al., 2007). Similarly, deletion of Sen1 in *S. cerevisiae* causes transcription-associated DNA recombination defects, connected with transient accumulation of R-loop structures (Mischo et al., 2011). These findings suggest that R-loop formation is a frequent event during transcription and that Sen1 acts to prevent their accumulation and the associated genome instability events. Possibly, the tendency of transcription to induce R-loop formation is a general feature of all eukaryotic genomes, which requires a range of dedicated helicases to resolve these potentially harmful structures.

Recently, senataxin was shown to be involved in splicing regulation (Suraweera et al., 2009). This might be related to the defect in Pol II elongation or defects in interaction with RNA splicing factors. Interestingly, previous studies demonstrated that defects in splicing induced by the depletion of SR splicing factor ASF/SF2 correlate with increased R-loop levels, resulting in genome instability (Li and Manley, 2005). In addition, capping enzyme was shown to promote R-loop formation (Kaneko et al., 2007a). Mutations in senataxin protein cause AOA2/ALS4 neurodegenerative disorders. Even though senataxin is ubiquitously expressed, neuronal genes display high levels of alternative splicing. Possibly, defects in senataxin function are more severely manifested in neuronal tissues. As compared to yeast Sen1, which has exclusively nuclear localization, senataxin is also detected in the cytoplasm, where it may have additional functions. Future structural studies on this protein and the analysis of its cytoplasmic roles may further clarify the role of senataxin in human cells.

## Experimental Procedures

### Cell Culture Analysis

HIV promoter-driven β-globin/MAZ_4_, mMAZ_4_, MAZ_4_ΔpA, ΔCoTC, and β-globin/β-actin plasmids were cotransfected with TAT plasmid into HeLa cells (except in the experiment illustrated in Figure 2B, where TAT was omitted) as described previously (Dye and Proudfoot, 1999; Gromak et al., 2006; West et al., 2004). GFP-RNase H1 plasmid was a kind gift from R.J. Crouch (Cerritelli et al., 2003). The RNAi procedure was carried out as described (Wollerton et al., 2004) (Supplemental Experimental Procedures).

### RNA Analysis

Total RNA was isolated using TRIzol reagent (Invitrogen) and reverse transcribed with SuperScript III Reverse Transcriptase (Invitrogen), using gene-specific reverse primers (Table S1). Spliced β-globin mRNA was detected with ex1(F)/ex3(R) primers. RNase protection analysis employed a riboprobe spanning the HIV transcription start site (−161 to +123) (Ashe et al., 1995; Plant et al., 2005). The termination efficiency of the β-globin-based gene constructs was calculated as a ratio between readthrough transcript A (AF/AR primers) and correctly initiated transcript B (intr1-ex2(F)/ex2(R) primers), using qRT-PCR analysis. NRO procedure is described in Supplemental Experimental Procedures.

### ChIP Analysis

ChIP was carried out using anti-senataxin (Bethyl Laboratories, Inc.), anti-Pol II (H-224) (Santa Cruz Biotechnology, Inc.), and anti-Xrn2 (Gromak et al., 2006) antibodies as previously described (Kadener et al., 2002; West et al., 2004). The immunoprecipitated, nonprecipitated, and input DNAs were used as templates for qPCR, containing QuantiTect SYBR Green PCR Master Mix (QIAGEN) and primers (Table S1).

### DIP Analysis

DIP was performed without a crosslinking step, following the ChIP protocol with some modifications. After the nuclear lysis reaction, extracts were incubated with 30 μg of proteinase K (Roche) at 55°C for 3 hr, and genomic DNA was isolated. Following sonication, DIP analysis was carried out using an antibody that recognized RNA/DNA hybrids, purified from S9.6 hybridoma cell lines (Boguslawski et al., 1986). The immunoprecipitated, nonprecipitated, and input DNAs were used as templates for qPCR. DIP RNase-sensitivity analysis was carried out by adding 200 U of S1 Nuclease (Fermentas) and 10 U of RNase H (NEB) prior to the immunoprecipitation step. The 100 μl nuclease digestion reaction contained 10× reaction buffer, and was performed for 2 hr at 37°C.

## Supplemental Information

Document S1. Two Figures, One Table, Supplemental Experimental Procedures, and Supplemental References
