# Supplementary material for: Human Senataxin Resolves RNA/DNA Hybrids Formed at Transcriptional Pause Sites to Promote Xrn2-Dependent Termination
Source: Mol Cell. 2011 Jun 24;42(6):794–805. doi: 10.1016/j.molcel.2011.04.026 (PMC3145960; doi:10.1016/j.molcel.2011.04.026)
Supplement: Document S1. Two Figures, One Table, Supplemental Experimental Procedures, and Supplemental References [file mmc1.pdf]

Supplemental Information

Molecular Cell, Volume *42*

Human Senataxin Resolves RNA/DNA Hybrids

Formed at Transcriptional Pause Sites

to Promote Xrn2-Dependent Termination

Konstantina Skourti-Stathaki, Nicholas J. Proudfoot, and Natalia Gromak

A qRT-PCR

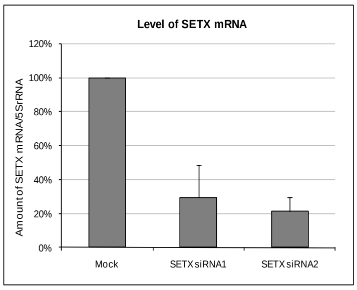

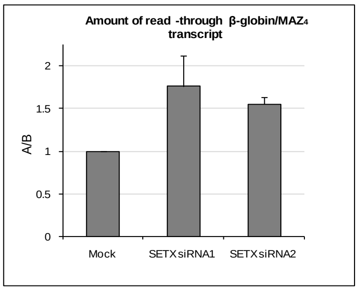

B RNase protection analysis

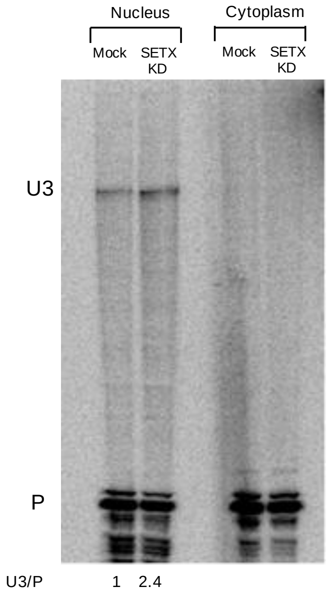

Figure S1, Related to Figure 1. Transcriptional Termination Defect of β-Globin/MAZ_4 Construct in HeLa Cells Treated with SETX siRNA 2

(A) RNAi-mediated depletion of human senataxin in HeLa cells using SETX siRNA 1 and 2. <u>Left panel</u>: qRT-PCR analysis of mock- and senataxin siRNA-treated HeLa cells. Bars represent average values +/- SD from three independent biological experiments. 5S rRNA was used as an internal control. The amount of SETX mRNA in mock cells was taken as 100%. <u>Right panel</u>: Termination efficiency of β-globin/MAZ_4 construct from mock-treated and cells treated with SETX siRNA1 and siRNA2, using qRT-PCR analysis on the total RNA. Amount of read-through transcript was calculated as a ratio of RT-PCR product A, representing read-through RNA, vs RT-PCR product B, representing the level of efficient transcriptional initiation.The A/B ratio in mock-treated cells was taken as 1. Bars represent average values +/- SD from three independent biological experiments.

(B) RNase protection analysis of β-globin/MAZ_4 construct from mock-treated and HeLa cells treated with SETX siRNA 2, performed with P/U3 riboprobe (see Figure 1 for probe position). Correct transcription

initiation from the HIV promoter results in the protection of an 85 nt fragment (P) of the riboprobe. Read-through transcription results in full protection of the riboprobe giving a ~240nt band (U3). Enrichment of read-through transcripts in SETX-depleted cells as compared to mock-treated cells was calculated as a signal ratio of U3/P (right panel). U3/P ratio in mock-treated cells was taken as 1.

A

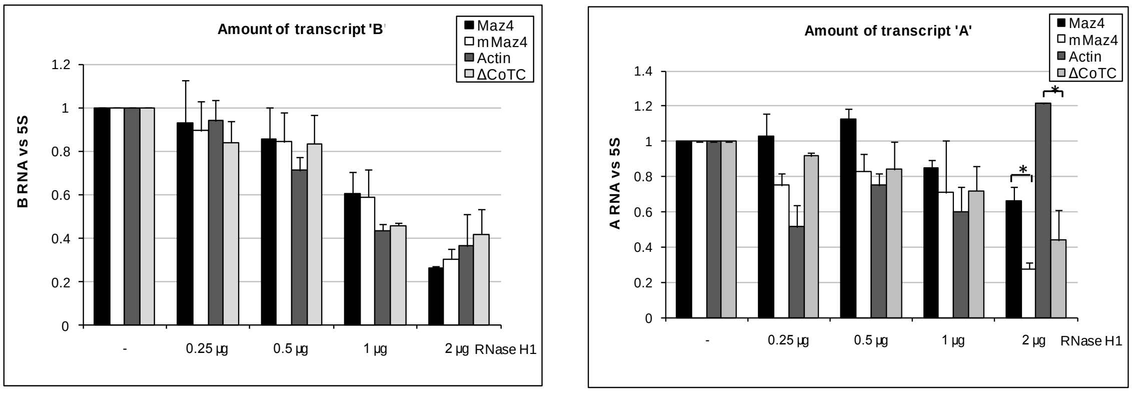

Figure S2, Related to Figure 3. RNase H1 Promotes Transcriptional Read-Through

Amount of correctly initiated (amplicon B, left panel) and read-through (amplicon A, right panel) β-globin/β-actin, β-globin/MAZ4, β-globin/mMAZ4 and ΔCoTC transcripts in mock- cells (sample -) and HeLa cells, cotransfected with increasing amounts of GFP-RNase H1 plasmid. The absolute amount of transcripts detected was normalized to the amount of 5S rRNA. Average qRT-PCR values +/- SD from four independent biological experiments are presented. The amount of transcript B in mock-treated cells (sample -) was taken as 1. * indicates significance ($p<0.05$), based on unpaired, two-tailed distribution Student's *t* -test.

It should be mentioned that the steady state transcript levels detected for A and B may be influenced by RNA degradation through RNAse H1 treatment. We therefore employed nuclear run on analysis as an independent assay to measure nascent read-through transcription, which is unaffected by RNA turn-over (Figure 3C).

Table S1, Related to Experimental Procedures. Oligonucleotide Sequences

**Name**	**Sequence (5′ ⟶ 3′)**
**β-Actin Gene**	
prom (F)	CCG AAA GTT GCC TTT TAT GGC
prom (R)	CAA AGG CGA GGC TCT GTG C
in1 (F)	CGG GGT CTT TGT CTG AGC
in1 (R)	CAG TTA GCG CCC AAA GGA C
in3 (F)	TAA CAC TGG CTC GTG TGA CAA
in3 (R)	AAG TGC AAA GAA CAC GGC TAA
in5 (F),	GGA GCT GTC ACA TCC AGG GTC
in5 (R)	TGC CAC TGG CTC GTG TGA CAA
5’pause (F)	TTA CCC AGA GTG CAG GTG TG
5’pause (R)	CCC CAA TAA GCA GGA ACA GA
pause (F)	GGG ACT ATT TGG GGG TGT CT
pause (R)	TCC CAT AGG TGA AGG CAA AG
C (F)	TGG GCC ACT TAA TCA TTC AAC
C (R)	CCT CAC TTC CAG ACT GAC AGC
D (F)	CAG TGG TGT GGT GTG ATC TTG
D (R)	GGC AAA ACC CTG TAT CTG TGA
F (F)	CCA TCA CGT CCA GCC TAT TT
F (R)	TGT GTG AGT CCA GGA GTT GG
**β-Globin Based Constructs**	
5’ ex1 (F)	GAG TTA GCT CAC TCA TTA GGC
5’ ex1 (R)	CTG TGT GAA ATT GTT ATC CGC
ex1 (F)	GAA CCC ACT GCT TAA GCC TC
ex1 (R)	CCT CAC CAC CAA CTT CAT CC
ex2 (F)	TTG GAC CCA GAG GTT CTT TG
ex2 (R)	GAG CCA GGC CAT CAC TAA AG
ex3 (F)	AGC TCC TGG GCA ACG TGC TGG
ex3 (R)	TTG TGG GCC AGG GCA TTA GCC
3’pA (F)	AAA AGG GAA TGT GGG AGG TC
3’pA (R)	AGC CTC ACC TTC TTT CAT GG
5’pause (F)	CTG CAA ACA GCT AAT GCA CA
5’pause (R)	TGA ATC CTT TTC TGA GGG ATG
pause (F)	GGG ACT ATT TGG GGG TGT CT
pause (R)	GTT TTC CCA GTC ACG ACG TT
3’MAZ (F)	GGA AAC TAT TAC TCA AAG GG
3’MAZ (R)	GTT TTC CCA GTC ACG ACG TT
A (F)	TTG CCT TCC TGT TTT TGC TC
A (R)	CCG CTG TTG AGA TCC AGT TC
intr1-ex2 (F)	GGA GAC CAA TAG AAA CTG GGC
**5S rRNA**	
5S (F)	AGC GTC TAC GGC CAT ACC
5S (R)	GGT ATT CCC AGG CGG TCT C
**SETX**	
SETX (F)	CTT CAT CCT CGG ACA TTT GAG
SETX (R)	TTA ATA ATG GCA CCA CGC TTC

Supplemental Experimental Procedures

Nuclear Run-On Analysis

NRO procedure and positions of the NRO probes were described previously (Ashe et al., 1997; Dye and Proudfoot, 1999; West et al., 2004). The Br-UTP NRO was carried out largely as described (Lin et al., 2008) with some modifications. Nuclear pellets were resuspended in Transcription Buffer (40 mM Tris-HCl pH 7.9, 300 mM KCl, 10mM $MgCl_2$, 40% glycerol, 2 mM DTT) and 10 mM mix of rATP, rCTP, rGTP, and Br-UTP or rUTP (in the control samples). The NRO reaction was performed at 30°C for 30 min. Total RNA was isolated using TRIzol reageant (Invitrogen) according to manufacturers' instructions and treated with RNase-free DNase I (Roche). 2 μl of anti-BrU antibody (Sigma) were pre-incubated with 30 μl of Protein G Dynabeads (Upstate) and 10 μg tRNA per sample for 1 hr at 4°. The beads were washed three times with RSB-100 buffer (10 mM Tris-HCl pH 7.4, 100 mM NaCl, 2.5 mM $MgCl_2$, 0.4% Triton X-100) and resuspended in 150μl RSB-100 with 40U RNase-OUT (Invitrogen) and 5μg of glycogen. Total RNA was added to the beads and incubated for additional 1 hr at 4°C. Then the beads were washed three times with RSB-100 buffer. RNA-bound to the beads was extracted with TRIzol reagent followed by two DNase I treatments. The RT reaction was performed using SuperScript III Reverse Transcriptase (Invitrogen) following the manufacturers' instructions. The real-time quantitative PCR was performed using a Corbett Research Rotor-Gene GG-3000 machine. The PCR mixture contained QuantiTest SYBR green PCR master mix (QIAGEN), 2 μl of template cDNA, and primers from the Table 1. Cycling parameters were 95°C for 15 min, followed by 50 cycles of 95°C for 15 s, 58°C for 20 s, and 72°C for 20 s. Fluorescence intensities were plotted against the number of cycles by using an algorithm provided by the manufacturer. The data was presented as a fold of enrichment of BrU-RNA over the U-RNA produced over a specific probe.

Senataxin RNAi and Western Blot Analysis

mRNA target sequence for SETX siRNA1 was 5'-GCCAGAUCGUAUACAAUUA-3'(Dharmacon) and for SETX siRNA2 –5'-CAATGGGTGGAAGCCGTCGTCAAAT-3' (Invitrogen). Western blotting was performed on 60 μg of total HeLa cell protein extract with antibodies raised against senataxin (Bethyl Laboratories Inc.) at 1:1000 dilution, GFP (AbCam) at 1:2000 dilution and actin (Sigma) at 1:2000 dilution.

Supplemental References

Ashe, H.L., Monks, J., Wijgerde, M., Fraser, P., and Proudfoot, N.J. (1997). Intergenic transcription and transinduction of the human beta-globin locus. Genes Dev. *11*, 2494–2509.
